# The multi-actor approach in thematic networks for agriculture and forestry innovation

**DOI:** 10.1186/s40100-021-00209-0

**Published:** 2022-01-17

**Authors:** Elena Feo, Pieter Spanoghe, Els Berckmoes, Elodie Pascal, Rosa Mosquera-Losada, Alexander Opdebeeck, Sylvia Burssens

**Affiliations:** 1 Department of Plants and Crops, Laboratory of Crop Protection Chemistry, Ghent University, Coupure Links 653 Block B, 6th Floor, 9000 Ghent, Belgium; 2 Proefstation Voor de Groenteteelt VZW, Duffelsesteenweg 101, 2860 Sint-Katelijne-Waver, Belgium; 3 Chambre d’Agriculture de Région Ile de France, 19, Rue d’Anjou, 75008 Paris, France; 4 Department of Crop Production and Project Engineering, University of Santiago de Compostela, Praza do Obradoiro, 0, 15705 Santiago de Compostela, A Coruña Spain; 5 Research Coordination Office – EU Team, Ghent University, Campus Ufo, Rectorate, Sint-Pietersnieuwstraat 25, 9000 Ghent, Belgium

**Keywords:** Knowledge sharing, Co-creation, Innovation, EIP-AGRI, EURAKNOS, EUREKA

## Abstract

The co-creation and sharing of knowledge among different types of actors with complementary expertise is known as the Multi-Actor Approach (MAA). This paper presents how Horizon2020 Thematic-Networks (TNs) deal with the MAA and put forward best practices during the different project phases, based on the results of a desktop study, interviews, surveys and expert workshops. The study shows that not all types of actors are equally involved in TN consortia and participatory activities, meaning TNs might be not sufficiently demand-driven and the uptake of the results is not optimal. Facilitators are key to contributing to the relationships and the mutual understanding between different actors. Moreover, a user-friendly digital knowledge platform linked to demonstration activities and peer-to-peer exchange can improve the sharing of knowledge, enhancing impact in agricultural and forestry innovation in the longer term.

**Supplementary Information:**

The online version contains supplementary material available at 10.1186/s40100-021-00209-0.

## Introduction

Co-creating solutions and sharing knowledge between different actors that have complementary expertise are key to promoting innovation in agriculture and forestry. According to the European Commission, innovation is defined as the introduction of something new which turns into an economic, social or environmental benefit for rural practice (COM 2014). Innovation becomes a nonlinear and iterative learning process with intense collaboration between different actors when solutions are co-created (Frow et al. 2015; Lundsgaarde and Keijzer 2019). Collaboration gives the possibility to share ideas and turn existing knowledge and research results into innovative solutions that can more easily be put into practice. This approach is also known as the Multi-Actor Approach (MAA) and represents the actor’s joint forces in project activities, from the conceptualization to the post-execution phase. Additionally, according to Brunori et al. (2020) and Schwarz et al. (2021), the interaction and co-creation among actors are key elements for a trans-disciplinary approach that have the potential to address future challenges in the forestry and agri-food systems. Moreover, it may help actors to develop new competences and skills that enable them to take on the roles of different types of actors (e.g. change agent, intermediary, knowledge broker and capacity builder). In the context of Horizon 2020 EU projects, an “actor” is a partner taking an active part in project activities, while a “participant” is a person expressing a view/stake at a certain moment during the project. On the other hand, a “user” is a person that concretely uses and benefits from project outputs, he/she can be both an actor and a participant. Actors, therefore, take an active role within a project, influencing its direction and outcomes. Participants, although they have a stake in the outcomes of the project, do not necessarily invest time and energy in the collaborative process.

The European Commission promotes and facilitates the actors’ interconnection through the Agricultural European Innovation Partnership (EIP-AGRI). The EIP-AGRI aims to bridge the gap between research and practice using the MAA, in line with the Agricultural Knowledge and Innovation Systems (AKIS) principles (COM 2012; COM 2013, SCAR WG AKIS 2019). The MAA is implemented through different types of projects related to agriculture and forestry as an “interactive innovation model”. In these projects, innovative ideas can be further developed into products and services by bringing together all the relevant actors at regional, national and EU levels (Van Oost et al. 2017). In the MAA, project users are directly involved in the development process of the results. Thanks to the transdisciplinary work of complementary actors, knowledge and solutions for daily needs at the field level are created together with farmers and foresters (Contini et al. 2020). The MAA not only ensures the active participation of project consortium members but also connects external actors.

The EIP-AGRI puts into practice and implements the MAA through Operational Groups (OGs), Multi-Actor Projects (MAPs) and Thematic Networks (TNs). Table 1 describes their characteristics and their goal.

**Table 1:** The application of the MAA in the EIP-AGRI

Application of the MAA in the EIP-AGRI	
Tools	Characteristics
Operational groups (OGs)	Funded by the EIP-AGRI, the formation of OGs should take place on the initiative of actors willing to face together a common problem or need on their work. All partners in the OGs have an active role in carrying out the innovative project. OGs have to draw up a plan that describes their specific project and the expected results. Furthermore, they have to disseminate the results of their project, in particular through the EIP-AGRI network (COM 2020)
Multi actor projects (MAPs)	Horizon 2020 (H2020) funded projects to develop innovative solutions which are ready to be applied in practice and cover real needs
• Research and innovation actions (RIAs)	Aim: establish new knowledge by exploring the feasibility of a new technology applied on a small scale
• Innovation actions (IAs)	Aim: producing plans and arrangements for prototyping, testing, demonstrating, large-scale product validation and market replication
• Coordination and support actions (CSAs)	Aim: achieve measures such as dissemination and communication, awareness-raising, networking, support services, policy dialogues and mutual learning exercises for new infrastructure and complementary activities
Thematic networks (TNs)	Type of CSA project. TNs disclose existing knowledge and best practices in a given agriculture and forestry theme (EIP-AGRI 2016). Their results and outputs are oriented to compile, produce and share innovative ready-for-practice solutions in easily understandable formats for users (e.g. farmers, foresters and advisors). They follow a bottom-up approach, taking into account farmers’ and forester’s experiences and sustaining them with scientific knowledge (Curry and Kirwan 2014; SCAR WG AKIS 2019)
Common goal: the potential to transfer knowledge and innovative solutions at regional and national levels. The innovative solutions can be implemented by actions funded by the Common Agricultural Policy (CAP) in the Member States (Matthews 2020). The results achieved through the application of the MAA can ultimately lead to changes in the allocation of public resources, policies, and regulations (Gullino and Vivani, 2021)	

This study focuses on providing guidelines to maximise the MAA in TNs, and it was performed within the H2020 EURAKNOS project.^1^ EURAKNOS stimulates the exchange of existing approaches, methodologies and tools between TNs. Additionally, it searches for a harmonised approach for setting up future TNs to maximise the MAA, as well as the TNs’ impact on their users. This project also explores users’ needs and the possibility of setting up an open-source European agricultural knowledge and innovation system that connects all TNs, enhancing the knowledge exchange. The European Commission calls on the Member States to take advantage of the potential of such platforms in the agriculture and forestry context. This is necessary to achieve the objectives set by the current and future Common Agricultural Policy (CAP), where knowledge exchange and digitalization are present in the general objectives, in the specific objectives, in the chapter on knowledge systems in agriculture and the chapter on agricultural modernization. Additionally, the use of transdisciplinary platforms may have a positive impact upon the direction of research that can transform farming, forestry and food systems at EU and national levels (Schwarz et al. 2021).

Despite the effort that the EIP-AGRI is making to implement the MAA, the concept is not easily applicable due to a vast heterogeneity of actors and different ways of interaction in projects (Macken-Walsh 2019). As such, the MAA and the interactive innovation model are not always applied to their full potential. As a consequence, farmers and foresters are often not aware of the existence of MAPs and their outputs (Šūmane et al. 2018). As one of the main user groups, farmers and foresters have to be actively involved in project activities and help the transformation of their needs into knowledge and innovations, which can greatly benefit the project outcomes. Although strategies have been developed to facilitate the interactive process throughout the years (e.g. discussion groups, self-appraisal), the MAA remains a case-dependent concept (Klerkx et al. 2017; Macken-Walsh 2019). Thus, a uniform implementation model in which actors can follow specific guidelines does not yet exist (Macken-Walsh 2019).

The MAA is a relatively new concept and in recent years, several studies regarding the importance of this approach to enhance knowledge sharing were performed (Macken-Walsh 2016; Lundvall, 2016; Ingram et al. 2020). Additionally, in light of the transformation occurring in the agri-food sector (e.g. Agriculture 4.0), Rose et al., (2021) stated that “*a successful MAA, is one step towards determining a responsible course for the fourth agricultural revolution to ensure that benefits are provided for people, production, and the planet*” (Rose et al. 2021). Within the EU, the MAA in different types of collaborative projects was also studied within the frame of the LIAISON H2020 project.^2^ LIAISON reviewed the MAA performance of 200 co-innovation partnerships from across Europe (e.g. Interreg, LIFE, ERASMUS+), finding that many of the project consortia were composed by actors with past project experiences. Moreover, a lack of farmers and foresters involvement in the EIP-AGRI activities was noticed. If this continues in future projects, the success of individual multi-actor partnerships and the overall EIP-AGRI policy objective of ‘speeding up’ innovation may be jeopardised (Fieldsend et al. 2020; Fieldsend et al. 2021).

In general, despite the relevance of the existing studies on multi-actor partnerships in the EIP-AGRI, a thorough analysis of how TNs perform the MAA has never been conducted before. Hence, specific guidelines for this typology of projects are missing. To overcome the shortcomings of previous studies, this paper conducts a MAA study, shedding the light on best practices and pitfalls to avoid in future projects. As such, the study helps to speed up the innovation process and to enhance the interconnection of actors taking parts in different TNs*.* At the time of the analysis, 34 TNs were funded by Horizon 2020 of which 6 just had started and therefore, were not included in this study. Hence, the MAA study was conducted in the framework of 28 TNs.

The work is divided into two main research tasks. The first analysis investigates how consortia are composed and which types of actors are involved in these MAPs. The second analysis focuses on the engagement of the actors during the three phases of a TN project (i.e. conceptualisation, implementation and post-execution phase) (EURAKNOS 2020). In this way, this paper evaluates how to improve the interaction and knowledge sharing between the different actors, in particular with farmers and foresters.

The remainder of this paper is organized as follows. First, the methodology section describes the strategies used to address the research questions. The main findings of this paper are then presented and subsequently discussed. Finally, the main outcomes of this work and future prospects are presented in the conclusion section.

## Methodology

**Fig. 1:**
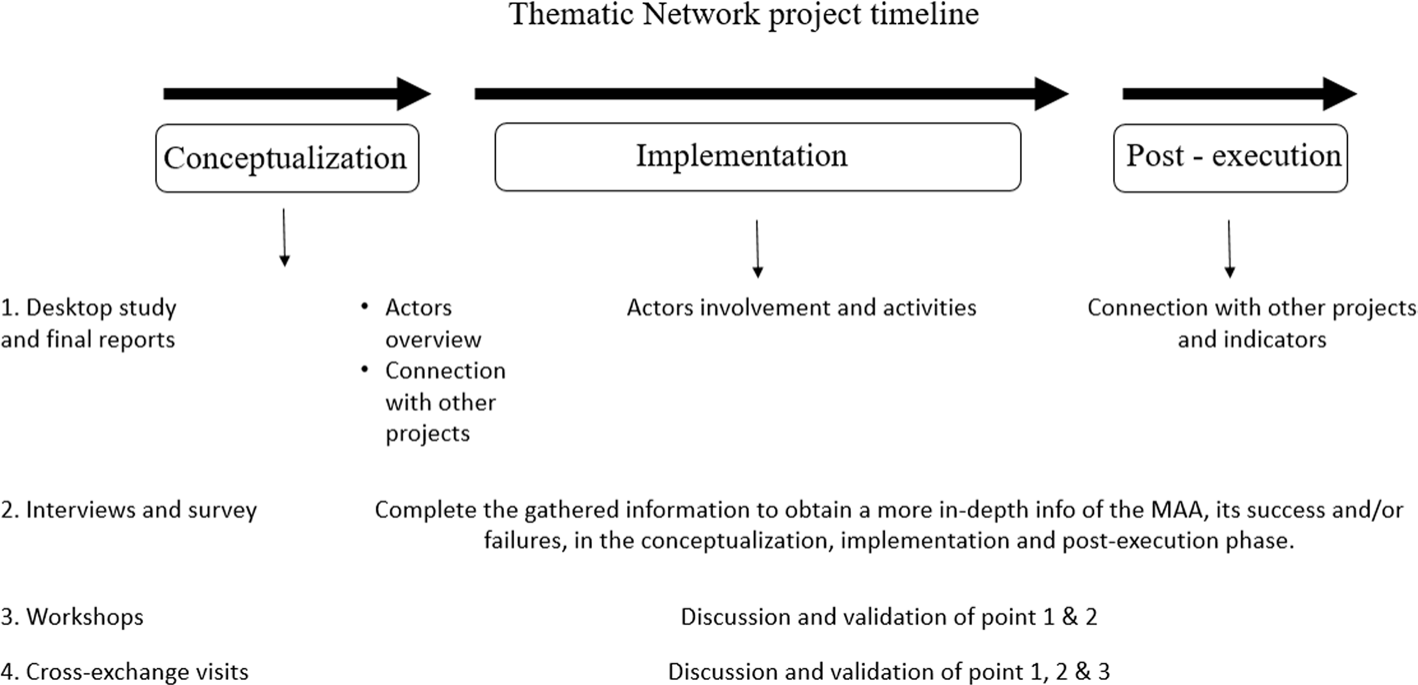
Experimental design of the work

The experimental design of this work consisted of several steps, as presented in Fig. 1. These steps were organised according to the three main phases in TN project management life cycle (i.e. the conceptualization, implementation and post-execution phase). First, a desktop study was performed on the website of all TNs to have an overview of the composition of the consortium (e.g. number, origin, and type of actors involved), as well as their connections with external projects (e.g. OGs, MAPs and other TNs). Second, to complete the information gathered with the desktop study, a series of interviews and a survey were performed to obtain a more in-depth overview of the MAA. Lastly, to validate and further discuss the previous steps, 3 participatory workshops and 6 cross-exchange visits (CEVs) were organised in order to have a complete overview of TNs’ tools and strategies for a functional MAA. Each of these steps are discussed in the following subsections.

### Identification of TN project phase

**Fig. 2:**
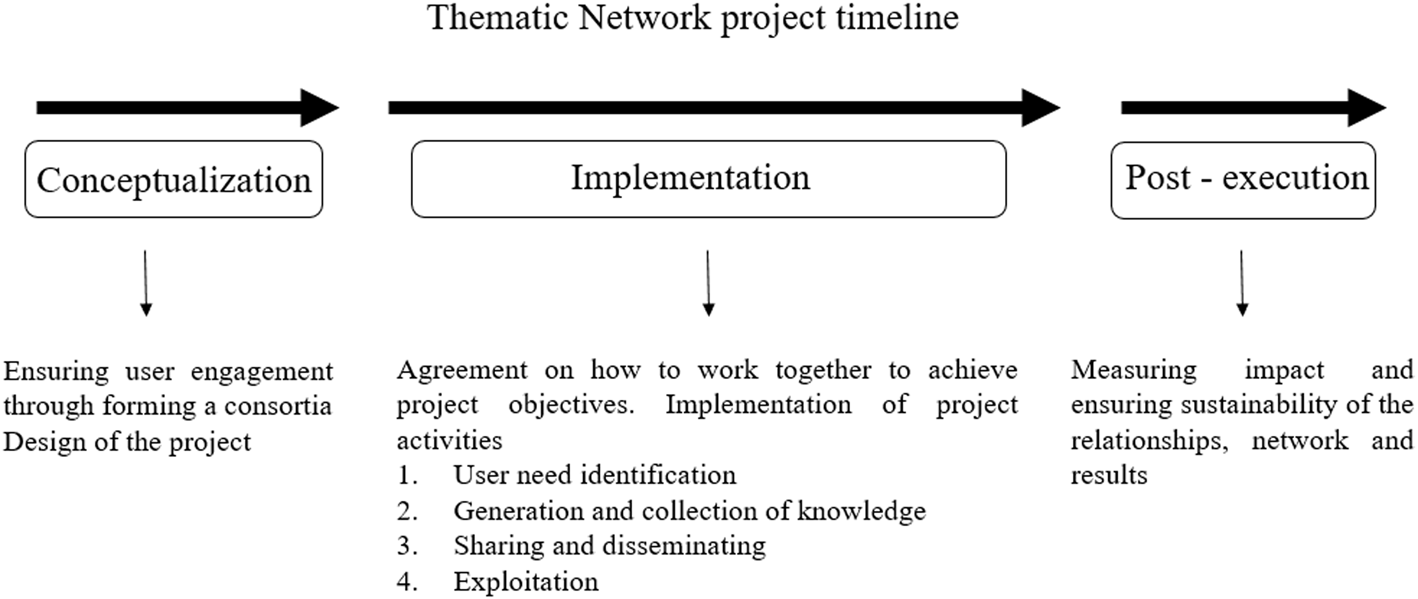
The TN project timeline: conceptualisation, implementation and post-execution phase (EURAKNOS expert workshop, December 2019)

The analysis of the MAA was performed considering three main phases of project writing and execution. We identified these as the following: (i) the conceptualisation or pre-award phase, (ii) the implementation or post-award phase and (iii) the post-execution phase. These are also shown in Fig. 2. Firstly, in the conceptualisation phase, the project concept is defined, the partnership is formed and the project implementation strategy is developed. Secondly, in the implementation phase, the consortium has to agree on how to work together to achieve the project objective. In this phase, the knowledge is co-produced and project results are communicated, disseminated and exploited by the target groups. Lastly, in the post-execution phase, the achievements of the project are analysed and the effective uptake of the outputs is evaluated.

After identifying the three phases, we used several tools and materials to assess the MAA in TNs. These are each discussed in the following subsections.

#### Desktop study: online evaluation of 28 TNs

All TNs that started since 2015 (34 TNs) were considered between January and March 2019. However, six TNs just started at the moment of the analysis and did not dispose of a website and/or sufficient data to be taken into account for this analysis’ purpose. Seventeen other TNs were in an advanced stage of project progress. The last eleven TNs were in their first year of project work. Thus, in total, 28 Horizon 2020 funded TNs were investigated in this research. These are listed in Table 3, along with their participation in each of the steps in our experimental design.

All collected data were publicly available on TN websites and the CORDIS (Community Research and Development Information Service) website. In the desktop study, five main aspects were investigated: (1) type of actors involved in the TN consortium, (2) residence country of TN consortium partners, (3) partner’s allocation budget, (4) connection with OGs and/or other MAPs, and (5) participatory MA activities (e.g. CEVs, workshops, field/farm days) in the conceptualisation, implementation and post-execution phase with the emphasis on user groups’ involvement.

Additionally, TN coordinators were asked to provide the mid-term and final reports of their project, for a total of 14 reports (Table 3). These reports were additionally consulted regarding methodologies and experiences used to develop a functional and effective MAA in the TN.

#### Interviews and survey

The face-to-face (F2F) interviews aimed to complete the information gathered with the desktop study to obtain a more in-depth overview of the MAA of the TNs, the implementation and its success and/or failures. The interviews took place between March and May 2019. The questions for the oral interviews were grouped as follows.

1. The actor's involvement and engagement in the TN;
2. The main contributor and the missing actor in the co-creation process of the TN;
3. Tools and strategies to enhance farmers engagement;
4. The barriers faced in a TN regarding the MAA;
5. The stimulation of the co-creative process in a TN.

The design of the F2F interviews combined with an online survey followed a three-step validation process. Firstly, a draft was performed and the questions were discussed, prioritised and circulated among all EURAKNOS project partners. Secondly, the first list of interview questions was corroborated by TNs coordinators involved in the project consortium (*SMART AKIS*, *Hennovation*, *OK-net Arable* and *Inno4Grass*). Thirdly, the questions were redrafted, an online survey considered all quantitative questions (61 in total); the qualitative questions (14 in total) were kept for the F2F interviews. For the online survey, the tool SurveyMonkey® was used. The complete questionnaire used during the interviews and the online surveys is given in Additional file 1: Appendix 1.

Twenty-seven interviews were carried out through physical meetings (16) and Skype calls (11). Interviews were performed with TNs key actors responsible for the MA involvement and TNs coordinators (Table 2). Since 13 TNs never replied to the online survey, the results are based on 15 out of 28 TNs.^3^ These TNs are reported in Table 3.

**Table 2:** Actors involved in the MAA investigation

Methodology	Experts involved	Number of participants
Desktop study	TNs website, CORDIS	28 consulted websites
F2F interviews	TN coordinators, MAA experts	27
Online survey	TN coordinators, MAA experts	15
Budapest workshop	18 TN coordinators, 8 advisors, SMEs, 5 farmers/foresters, 2 chambers of agriculture, 6 farmer organisations, 4 research organisations, 2 government authorities, 3 educational institutions, 5 universities, 2 NGOs	58
Paris workshop	1 TN coordinators, 2MAP coordinators, 2 researchers, 4 farmer/foresters, 2 advisors, 1 policymaker	12
Online workshop	14 TN coordinators, 17 advisors, 12 consultants, 8 policymakers, 16 researchers, 14 farmers/foresters, 6 SME, 2 IT expert	89
Cross exchange visits	18 TN coordinators, 15 vocational schools, 20 farmers/foresters, 12 farmer’s associations and 16 advisors	81
		Total: 278 consulted experts

**Table 3:** TNs involved in the MAA investigation

Types of tools and materials used for the analysis								
	Desktop study	Face-to-face interviews	Online survey	Final report	Workshops (Budapest)	Workshops (Paris)	Workshops (online)	Cross-exchange visit
*Thematic network*								
AgriSpin	x	x		x	x		x	x
HNV-Link	x	x						
SMART AKIS	x	x		x	x			x
Agriforvalor	x	x		x				
AFINET	x	x						
Inno4Grass	x	x			x	x	x	
SKIN	x	x	x	x	x		x	
Enabling	x	x	x		x			
Newbie	x	x	x	x	x		x	x
Suwanu Europe	x	x	x					
OK-net Arable	x	x	x	x	x		x	
OK-net Ecofeed	x	x	x		x		x	
FERTINNOWA	x	x	x	x	x		x	
Winetwork	x	x	x	x	x		x	x
EUFruit	x	x	x	x				
CERERE	x	x					x	
INCREDIBLE	x	x			x			
PANACEA	x	x						
INNOSETA	x	x						x
Best4Soil	x	x	x		x			
Legumes translated	x						x	
Nutriman	x	x	x		x			
Hennovation	x	x	x	x	x		x	x
EuroDairy	x	x	x	x				
4D4F	x	x	x	x	x		x	
EUPig	x	x			x		x	x
SheepNet	x	x		x	x		x	
Disarm	x	x	x	x	x			x
Total	28	27	15	14	18	1	14	8

#### Workshop

Three participatory workshops with TN actors were organised. During these workshops, the results obtained from the desktop study, F2F interviews and the online survey were further discussed and validated. The participatory workshops consisted of plenary sessions and parallel working sessions for which the group was split into subgroups. The parallel working sessions consisted of round table discussions where participants were asked to present their view on the specific aspect of the MAA listed below. A facilitator was in charge of moderating each working session. During the discussions, participants were asked to present their view and their opinions were collected and written down on a post-it board. In this way, they could be presented and further discussed during the plenary sessions.

The first workshop was held in September 2019 in Budapest. A total number of 58 participants from 17 Member States attended the event (Table 2). Participants represented the following categories of actors: advisors, SMEs, farmers/foresters, chambers of agriculture, farmer organisations, research organisations, government authorities, educational institutions, universities and NGOs. In total, 18 TNs were represented as reported in Table 3. The main points of discussion during the workshop were:

- Validation of the results obtained during the prior analysis of TNs (i.e. the desktop study, F2F interviews and online survey);
- Ways of communication of farmers and foresters on their needs in a TN;
- Type of potential actors/end users and ways of engagement of actors in TNs.

The second workshop was held in Paris in December 2019. Twelve experts from 7 Member States, of which researchers, farmer/foresters, advisors, and policymakers were involved. The TN *Inno4grass* and 3 others MAPs were represented (*SynSICRIS, FAIRshare* and *LIAISON*). The main objective from this workshop was:

- Discussion on best practice and methods to foster the MAA in TNs;
- Engagement of users in the conceptualisation, execution and post-execution phase of MAPs;
- Profile and role of a facilitator to catalyse the MAA process.

The third workshop was performed online in May 2020 through the use of the video conferencing tool Zoom (Version 5.4.9). The workshop was performed online due to the breakout of the COVID-19 pandemic.

The event gathered in total 89 participants (see Table 2 for detailed actor roles): partners and coordinators of the 15 TNs took part in the event, as reported in Table 3. Moreover, also actors involved in MAPs (*EUREKA, FAIRshare, I2Connect, NEFERTITI, SmartProtect, Organic PLUS, DESIRA*) and OGs (*CORE-ORGANIC, PLAID, Roadmap*) took part.

The main objectives from the online participatory workshop were to brainstorm on best practices for TNs, in particular on:

- Defining the roles of different TNs actors;
- Understanding user’s needs and identifying the best practices for collecting share and co-creating user-friendly knowledge and innovative solution;
- Designing a TN user’s engagement strategy;
- Enhancing TNs impact and sustainability.

The participants were divided into four breakout rooms (used as a replacement of physical workshop subgroups). A facilitator was present in each breakout room. The below tools were used to ask questions and create an interactive atmosphere with participants. The online survey systems Mentimeter^4^ and PollEverywhere^5^ were used to ask participants their opinion on the specific objectives of the workshop. In addition, the application MURAL^6^ was used as a replacement for a post-it board/flip-over chart. These tools allowed collecting participants experiences and suggestions.

#### Cross-exchange visits (CEVs)

To further investigate the MAA, 6 cross-exchange visits (CEVs) were organised between actors from TNs and the broader EIP-AGRI community (MAPs and OGs). They were carried out online between June and September 2020 through the use of the video conferencing tool Zoom. A total of 81 participated in these CEVs (see Table 2 for detailed actor roles). Partners and coordinators of 8 TNs took part in the event, as reported in Table 3. Actors from TNs and MAPs (*NEFERTITI, SMARTPROTECT, OPTIMA, FAIRShare*) were also present among the participants.

The investigated aspects related to the MAA during the CEVs are the following:

- Best tools for fostering the peer-to-peer exchange of knowledge;
- Best ways to share the TN outputs with users (in particular farmers and advisors);
- Maximisation of knowledge exchange between actors.

A facilitator was present in each CEV. The online tools that were used to create an interactive atmosphere with participants were the same used in the online workshop.^7^ Once again, this allowed to directly ask participants’ opinions on the specific objectives of the CEVs.

## Results

In this section, the main results of the previously described methodology are described. The analysis of the MA involvement led to the following observations, organised according to the three phases of a TN project:

### Conceptualisation phase

#### Type of actors involved in TNs

**Fig. 3:**
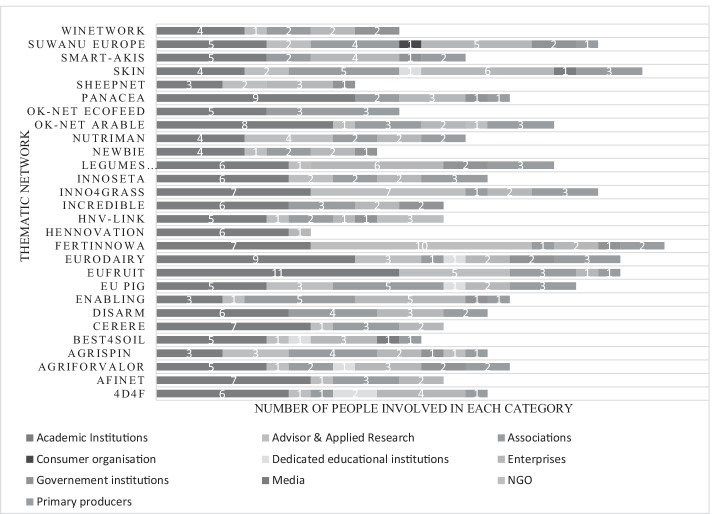
Composition of actors in the 28 TN communities based on the Desktop analysis

During the conceptualisation phase of a TN, it is decided which partners with which expertise will play a role in the consortium. The desktop study of the actors’ composition in 28 TN consortia showed that on average TN consortia are composed of 15 partners (Fig. 3). Academic institutions (i.e. universities) are present in all consortia. Other well-represented actor types are associations (farmers associations excluded) (present in 24 TNs), enterprises (present in 26 TNs), government institutions (present in 14 TNs) and, advisor and applied research (present in 22 TNs). On the other hand, rather underrepresented categories are: educational institutions (present in 5 TNs), consumer organizations (present in 1 TN), media (present in 2 TNs), and NGOs (present in 3 TNs), and primary producers or their representative organisations (present in 7 TNs) (Fig. 3).

During the F2F interviews, it was found that some key actors and/or expertise are sometimes missing in consortia. For example, the TN *FERTINNOWA* and O*k Net Arable* highlighted how these projects would have benefited from actors as sociologists, professional communicators, students from vocational schools and policymakers.

#### Geographical spread

**Fig. 4:**
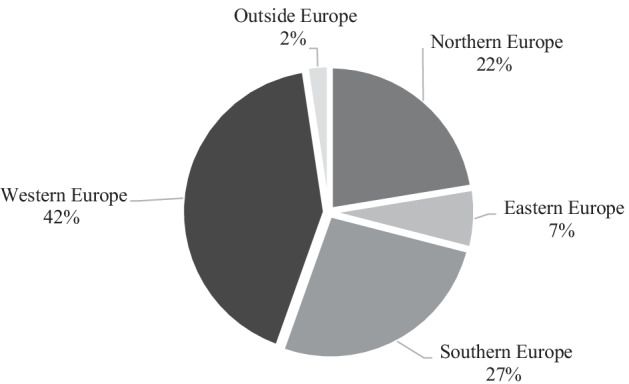
Geographical spread among different TNs in the EU (the result is based on the percentage of the total TNs (n = 28) granted during the years 2014–2019. Partner institutions were located in Western, Northern, Eastern, Southern and Outside Europe

The geographical spread among different TN actors is presented in Fig. 4. It shows the percentage of TNs and which part of the EU is most represented by actors in the consortia. The results derived from the desktop study were grouped into 5 regions: Northern, Eastern, Southern, Western and outside of Europe. Figure 3 shows that there is an inequality between Western (42%) and Eastern Europe (7%). This divergence is less evident in Northern (22%) and Southern countries (27%). Only 2% of TNs involved associated countries from the EU, such as Tunisia, Turkey, Ukraine and Norway, and there was one internal cooperation with South Africa. The most represented EU MS in TN consortia is Belgium (46 participations), followed by Spain (45 participation), France (43 participations) and Italy (37 participations) on a total of 300 institutions involved in TNs.

#### Demand-driven TNs

During the online survey, it was observed that 53% of the responding TNs considered themselves demand-driven. In contrast to this 53%, 27% of the TN projects are research and policy-driven. 13% of the TNs stated that their project was both demand and research/policy-driven and finally, 7% answered that their project was individual driven. These results tell us that a bottom-up approach, which is the basic reason for the existence of TNs, is well understood. Although, not all TNs are oriented towards users’ needs in practice.

**Fig. 5:**
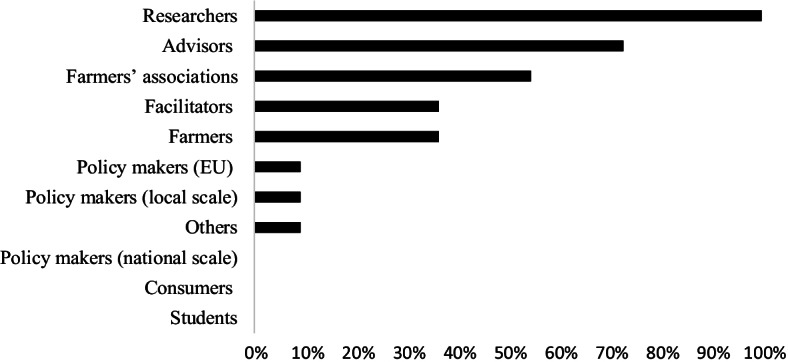
Percentage of actors involved in the conceptualisation phase of a TN (the percentage refers to the 15 answers from the online survey). Note that policymakers on a national scale, as well as consumers and students, are not involved (0%) in the conceptualisation phase

The F2F meetings with TNs coordinators and MAA representatives learned us that the type of actors taking up the responsibility in writing the proposal during the Conceptualisation phase is typically researchers (100%), followed by advisors (73%) and farmers associations (55%). Farmers and facilitators are less represented (36%). Consumers and students are not involved at all (0%) (Fig. 5).

From the F2F interviews, it was found that the main strategy to ensure the actor’s participation during Conceptualisation is to engage a well-defined target group of actors through being part of the consortium (87.5% of TNs). In co-creation, the scope and objectives of the project proposal are written. Regarding the involvement of farmers/foresters, TNs indicated that it is important to have (a) representative (s) as part of the consortium, or to have key intermediaries with a specific advisory role (e.g. advisors or applied research organizations/institutions). The TNs *Inno4Grass* and *SmartAKIS* involved European farmer's representative's associations in their advisory boards. More specifically, they involved COPA COGECA (Committee of Professional Agricultural Organisations-General Confederation of Agricultural Cooperatives) and EUFRAS (European Forum for Agricultural and Rural Advisors Services), respectively. Advisory Board members at a local, national and/or EU level meet less frequently during the project implementation and are mostly not directly involved in project activities. Additionally, the Advisory Board of *FERTINNOWA*, *CERERE* and *AFINET* directly involved users such as growers, consumer and government organizations, as well as industry and NGOs.

#### Facilitation of TNs

During the workshops, participants stressed the importance of the facilitator’s role to stimulate the co-creation process and involve users’ groups and discuss their needs, from the early stages of the conceptualisation phase. To remain impartial and objective, the facilitator may be replaced by another actor (that takes the same facilitating role) during subsequent phases of the project. Once the implementation of the project starts, the facilitator starts building a trusting space to allow healthy organic interactions amongst the consortium partners as well as interactions with external actors.

Facilitators are expected to possess three main skills: organizational (e.g. organisation of participatory workshops, webinars and discussion groups), social (e.g. creation of constructive dialogues, ability to attribute roles, being impartial, self-confident and sensitive to the needs of different actors, facilitation of networking and connecting skills of actors, bringing in new actors timely, being open-minded, flexible, creative, poly-math and empathetic, knowing about conflict management and having negotiating skills) and communicative skills (e.g. speaking in a local language, applying a user-friendly language with clear and conveying key messages).

The results collected during the consultation of the experts in the workshops confirmed that the involvement of facilitators in innovative agricultural projects is crucial to the success of MAA. Furthermore, it was agreed that facilitators should not necessarily be advisors. Any actor can cover the role as long as proper training is followed and the required skills are possessed. This viewpoint was supported by the TNs *Hennovation* and *Agrispin*, where facilitators helped the consortia and other external actors to detect problems and to set up new ideas.

#### Challenges of TNs during the conceptualisation

Other major challenges and possible solutions related to the Conceptualisation phase were identified during discussions in workshops at Paris, Budapest and online, and also during the cross-exchange visits. The main challenges and corresponding solutions are grouped in Table 4.

**Table 4:** Major challenges and possible solutions in TN's Conceptualisation phase

Challenges	Proposed solutions
Involve key actors and/or expertise in TNs consortium	Open to contact unfamiliar actors with previous project expertise
Improve geographic countries’ participation in TNs consortium	EC strategy is needed to balance the participation of the different regions in TNs
Target farmers and foresters’ needs in the project’s objectives	Increase the involvement of farmers/foresters and their associations through participatory activities
Improve the communication between researchers and farmers	Set up a common project activities agenda between researchers and farmers
Increase the involvement of educational institutions and students	Plan an agenda with common project activities together with educational institutions
Create a close-knit consortium	Organise team-building activities with the help of a facilitator
	Assign roles and workload depending on partners abilities
Select skilled facilitators	Set a methodology with selection criteria based on the organizational, social and communicative skills of facilitators

### Implementation phase

In this phase, the interaction among actors is explored once the consortium is formed. It describes how external actors are engaged in TNs.

#### Meeting frequency

The online survey indicated that the majority of TN consortia organise two physical meetings a year. This meeting frequency was perceived by 81% of the interviewed TNs as sufficient to co-create and to fulfil the project objectives. However, according to several coordinators, this frequency should be higher. Interviews pointed out that having regular yearly meetings increase the willingness to cooperate among actors. Furthermore, a clear communication strategy in the consortia regarding the meeting frequency need to be planned to increase the trust among actors.

#### Engagement of actors

The desktop study showed that the engagement of actors external to the consortia is mostly restricted to events such as workshops, CEVs, on-farm demonstrations, study days and focus groups. The TN *FERTINNOWA* and *Sheepnet* carried out short surveys and study meetings from the implementation phase to define the needs and bottlenecks of actors, using a bottom-up approach. The results were further addressed in a subsequent participatory workshop that ensured full partner engagement during the whole duration of the project. Similarly, the TN *Agrispin* used a storytelling strategy to learn about actors’ experiences as input for the project.

**Fig. 6:**
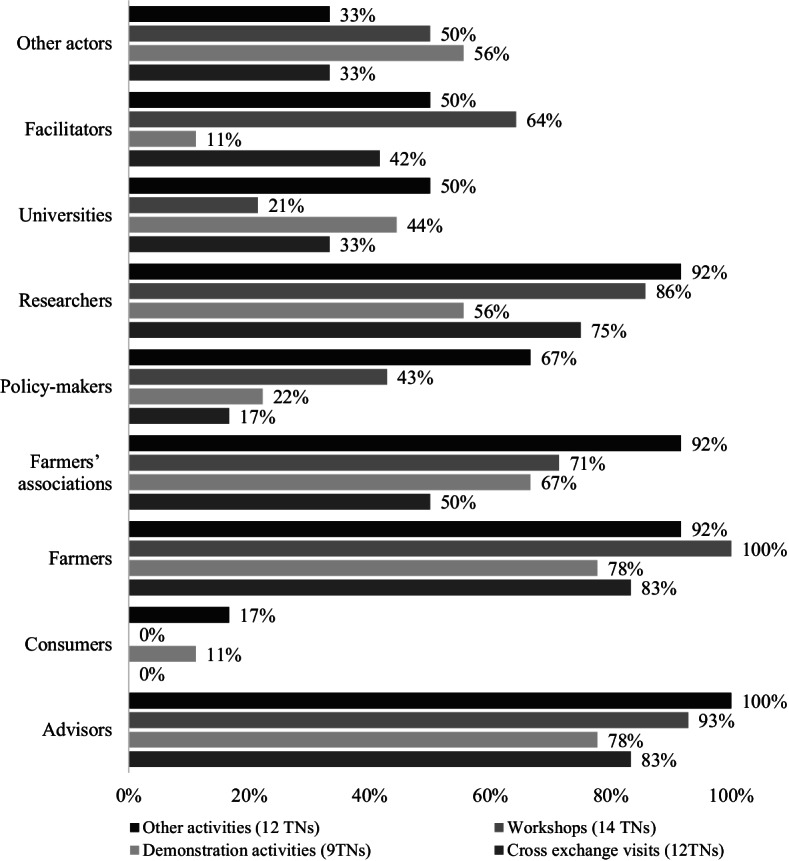
Percentage of different types of actors involved in TN activities. The result from 15 answers in the online survey. Note that consumers organization resulted in 0% since they were not involved in project workshops and cross-exchange Visits

Figure 6 shows the results about the engagement of the different actors in their project activities (e.g. workshops, field and demonstration days, CEVs) in the online survey. The category “Other actors” refers to, e.g. NGOs, SMEs, Umbrella organisations, and the category “Other activities” refers to, e.g. conferences, study days and training. The responses showed that farmers and advisors were the primary groups of actors taking part, as well as farmers’ organisations. Active participation of policymakers was observed in CEVs, demonstrations events, and MA workshops as well as “other activities”. Consumers and consumer organisations were found to participate during demonstration activities and “other activities”.

Despite the high percentage of farmers involved in participatory activities (Fig. 6), the TN coordinators highlighted during the face-to-face interviews that engaging farmers remains challenging as they do not wish to move across Europe to meet other farmers. They also pointed out that farmers usually work within their local network consisting of other farmers, advisors and other key intermediaries. Hence, reaching and involving them in TN activities is less easy. This observation was also put on the table during the discussions of the experts in the EURAKNOS workshops.

**Fig. 7:**
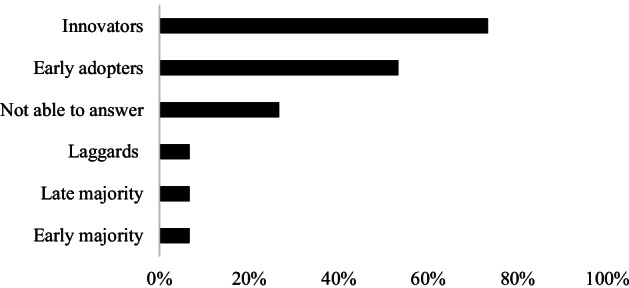
Type of farmers and foresters involved in TNs activities. The result from 15 answers in the TN online survey

Innovations are not adopted by all individuals in a social system at the same time. Instead, they tend to adopt in a time sequence and can be classified into adopter categories^8^ based upon how long it takes for them to begin using the new idea. From the online survey, out of the 15 responding TNs, 4 indicated not being able to specify the type of farmers and foresters involved in their TN (Fig. 6). Based on the 15 responding TNs, it was shown that farmers and foresters mainly involved in TNs activities are “innovators” (73%) and “early adopters” (53%). Only 1 TN, *Hennovation*, indicated that also the early and late majority and the laggards towards regional practice-led innovation networks were reached (Fig. 7).

An example of successful collaboration between actors from different countries is given by the TN *INCREDIBLE*. This TN created an “Interregional Innovation Networks” platform (iNets) to implement an innovation-driven knowledge sharing process. In such an innovation platform, actors from different European areas were brought together to exchange and discuss specific topics, solutions, and best practices. Furthermore, a dedicated space inside the online platform allowed iNets facilitators to collect and share experiences and discuss future approaches.

#### Challenges of TNs during the implementation

During discussions held in the expert workshops and during the cross-exchange visits, additional new insights emerged related to interaction among actors in the implementation phase. Major challenges and possible solutions were identified. The challenges are grouped in Table 5.

**Table 5:** Major challenges and possible solutions in TN’s implementation phase

Challenges	Proposed solutions
Alignment of different actors towards the same goal	Take partners profiles (background and expertise) into account
	Keep the focus on the specific TN challenges by having regular meetings and updates
	Communicate transparently between consortium partners
Uptake of TN results into practice	Involve a facilitator, create a space of trust where actors can co-exist
	Adopt a clear language easily understandable for users, avoid buzz words
	Distribute videos and podcasts through social media to give visibility to TN stories and best practices
	Translate dissemination materials in as many languages as possible
Participation of farmers, foresters and advisors	Use awards (e.g. *Newbie* gives yearly awards for farmers)
	Provide the possibility reallocate budget to compensate the time of farmers/foresters and (private) advisors (subcontracting)
	Contact farmers/foresters directly
	Involve representative organizations or existing networks of farmers/foresters and OGs (e.g. *EuroDairy* experienced a good connection with farmers due to the involvement of 42 OGs)
	Engage members in the Advisory Board who can facilitate contacts with farmers/foresters
Use of expertise from actors outside the EU	Engage international partners in the consortium and/or invite international experts to participatory workshops (e.g. *FERTINNOWA* and *Sheepnet* involved actors outside the EU in transnational workshops)
Improvement of the interaction between actors	Organise discussion groups or events where scientists and farmers can meet (e.g. *OK net Ecofeed* organised “Science Bazaars”)
	Involve small peer groups and incentivise activities that stimulate peer-to-peer learning among farmers/foresters and advisors
Involvement of experienced facilitators	Provide training for improving the facilitator’s softs skills
	Involve facilitators from the conceptualisation phase
Integration of TN results into practice	Create an open-access digital platform that houses the TNs outputs organised in a common easy language with defined concepts. The TNs *INCREDIBLE*, *SKIN*, and *4D4F* created an interactive knowledge platform

### Post execution phase

When the interviewees were asked how to ensure the sustainability of the network and its results, 3 TNs (11%) could not answer this question as the project was just started, 24 of the interviewed TNs (89%) mentioned the links with existing OGs, other TNs and user groups such as farmers’, foresters’ and advisors’ groups. Other answers referred to the importance of creating synergies between ongoing and follow-up projects and having easily accessible outputs for practitioners including the TN website. TNs final reports also showed that the evaluation of the “success” of the project and the short-medium and long-term impact of technology produced documents was made through the use of google analytics.

The number of views can indicate whether it is still useful to keep the website running after the project has ended. As the downloads of the main outcomes of *FERTINNOWA* 10 months after the end of the project were still high, it was decided to keep the website ongoing.

Major challenges and possible solutions related to impact evaluation in the post-execution phase were identified during the discussions in the workshops. The challenges are grouped in Table 6.

**Table 6:** Major challenges and possible solutions in TN's post-execution phase

Challenges	Proposed solutions
Ensuring the impact of TNs	Organize farm/field demonstrations involving OGs
	Reinforce network involving pilot farmers and experts across national/regional borders
	Translate the results into educational materials for courses, vocational schools and lifelong learning programs
	Establish a digital platform for communication and dissemination of project outcomes
	Establish a digital platform where users testimonies can be shared with peers
Accurate measurement of the impact of TNs	Use qualitative (proxy) impact indicators (e.g. number of users engaged in each activity)
	Use quantitative impact indicators (e.g. surveys, case studies, focus groups, interviews)
The organisation of financial resources (human resources, time, cost)	Perform the impact evaluation during new TNs/innovative agricultural projects taking into account the minimum period required for the uptake of results by users (3–5 years after the end of the project)
	Allocate funding schemes by national and regional departments

## Discussion

### Conceptualisation

From the composition of actors in the 28 TN communities (Fig. 2), it is noted that a well-balanced partnership among actors is not achieved. It is important that future TNs will work to equally represent all types of actors in the consortia, as confirmed in the SCAR AKIS (2019) report. The authors consider that the specific interest of actors should be taken into account, and the tasks should be assigned according to the actors’ abilities. However, this seems not always be the case in TN consortia (Fig. 2). Categories such as media, NGOs, consumers, educational institutions, primary producers and representative organisations (farmers/foresters) are rather underrepresented. Hence, the complementary expertise and sharing of knowledge that characterise the MAA are not working at their full potential. Moreover, the consortia might suffer from missing actors’ key skills and networks that can help to reach a wider audience and the TN’s success, in terms of uptake and implementation of the project results (Tables 5 and 6).

The lack of involvement of some actors in TN consortia can be due to the process of actor selection. Since this process can be strongly influenced by previous cooperation experiences, partners with previous project expertise are often valued more than new partners. This aspect was also highlighted in the study of Fieldsend et al. (2020). As such, the different experiences and tacit knowledge of these new partners are not used (Fieldsend et al. 2020).

Eastern, Southern and Northern European countries are less involved in TNs than Western European countries (Fig. 3). One reason that Von Münchhausen et al. (2019) pointed out could be due to cultural issues, which can affect actors engagement in the co-innovation projects. Users from all European countries should be addressed more equally and therefore, an effort may be undertaken at the EC level to keep the balance in the participation of the different regions in TNs. Specific measures that are coupled to the funding programme can be implemented as confirmed in a report of the EU Commission (COM 2017b).

Only half of the TNs examined in this study engaged user groups in the Conceptualisation phase (Fig. 2), meaning that they risk not translating sufficiently farmers and foresters’ needs in the project’s objectives. Having them involved in the consortia itself is hard due to the limited time and length of a TN project. The category of researchers is the most represented. The communication gap between researchers and farmers is an ongoing problem (FAO 2018). Hence, researchers should plan a common agenda with farmers that state and communicate clearly which are their expectations from the TNs. Furthermore, researchers can improve the way of communicating their ideas in an understandable way for farmers (Fieldsend et al. 2020). This ensures that scientific outcomes and practical knowledge are brought together to address the most urgent needs of users, as well as advanced ready-for-practice knowledge.

The observed outcome of this study suggested as one of the solutions that Farmers’ and foresters’ engagement can be strengthened through memberships in an Advisory Board and through participatory activities such as F2F interviews, workshops and surveys in the earlier stages of a TN. Furthermore, these pre-award participatory activities allow users and consortium members to take an active part in the shaping of the scope of the project in a co-creative process. They can also contribute to building trust between the partners and other key actors (Sewell et al. 2017).

Another category that is not enough targeted is the educational institutions, which often play an important role in stimulating the uptake of the results (Fig. 2). They do this through training initiatives such as vocational schools, lifelong learning programmes and advisory training, as demonstrated by Sewell et al. (2017). Hence, future TNs should make more effort to connect with those actors. Additionally, involving students from, e.g. vocational schools in the activities of TNs, allows them to take up the results and use these in the field. By providing them with more concrete experience, they are taken more seriously in decision-making processes. Moreover, based on the study of Coniavitis et al (2005), students’ can enhance practical and theoretical skills that they need to develop as potential future TN actors.

Partners contribution can be significantly improved, as this study showed, by implementing team building activities already at the beginning of the project. The authors suggest that all partners should agree on a “code of conduct” at the beginning of the TN, specifying how to work most effectively and how to keep up engagement throughout the lifetime of a TN, both inside and outside its consortium. A skilled and dynamic facilitator should be appointed to clearly define the complementary roles of each partner, streamline communication for effective teamwork, stimulate the co-creation process and engage the right actors at the right moment since the project beginning. (Madureira et al. 2019).

### Implementation

The authors concluded that the conceptualisation stage of the project is a crucial step that grants a good start to the implementation phase. The conceptualisation let to build trust and synergies that are necessary for the implementation phase. In the implementation stage, the project activities are further aligned to the needs of the users. Having an engagement strategy is necessary to optimise knowledge exchange and ensure uptake and exploitation of results.

As confirmed in the study of Calliera et al. (2021), workshops, CEVs, on-farm demonstrations, study days and focus groups are fundamental moments in a TNs’ life where actors share experiences and collaborate efficiently. It is important to involve local networks and OGs to facilitate the sharing of needs, and knowledge, and clarification of TN themes. In this way, it is easier to identify topics of interest and potential problems, to address them during the project, and to work together towards solutions, this is also confirmed by Macken-Walsh (2019). These activities allow the user groups to bring in their valuable practical experience, share tacit knowledge with their peers and other actors, as well as expand the knowledge in a broader geographical range. Several studies confirm as well that farmer-to-farmer interactions are major channels of knowledge sharing and innovation in the AKIS (Wielinga and Geerling-Eiff 2009; Rose et al. 2016). Even though Fieldsend et al. (2020) describe TNs as “among the leading exponents of stakeholder engagement ‘all along with the project”, our results showed that most of the time only farmers belonging to the categories of “Innovators” and “early adopters” were engaged in TNs, meaning that only a small segment of the active farmer community is reached. Nevertheless, the participation of farmers/foresters may be ensured and stimulated by providing financial compensation for their absence on the field. TNs should make them aware by showing concrete potential benefits if they are active actors, influencing the co-creation process instead of passive users.

In this phase, experts have pointed out that implementing a TN digital platform represents an important factor to strengthen the MAA. A digital platform that houses the TNs knowledge outputs should function so that the content is tailored to the profile, level of expertise and preferred language (automatic profiling and translation) of the visiting user. Ideally, having a digital platform that responds to each user creates trust, which can amplify the number of reached users and increase the impact. The TN *INCREDIBLE* and *Skin* developed an online platform for the implementation of a Virtual Community of Practice to support their existing network and expand it with new users, but also to exchange news and experiences and ensure the sustainability of the TN outputs. Moreover, the TN *Agriforvalor* uses its platform for storing educational and training materials. The observation regarding the digital platform that came out from this study pointed out that it should allow users that could not participate in events, to be informed of the results through videos or podcasts. Those materials should be translated into as many local languages as possible. Meetings in person are also needed, but the platform may represent and stimulate the curiosity of actors for future collaborations.

In line with AKIS strategies and the CAP, the creation of an EU-wide knowledge platform that collects all-ready-for-practice materials from all TNs will help to create an EU agriculture and forestry community, strengthening the MAA. Additionally, links between research, practice, education and advisors may be easily made and knowledge is potentially more efficiently exchanged outside the national borders. Even though these platforms are structured to serve TNs users’ needs, they can have an impact also at the political level. Policymakers can also close the gap between policy and practice, enhancing the effective use of TNs outputs. However, according to Burssens et al. (2020), adequate financial resources should be foreseen to maintain the platform over time.

In short, a knowledge database that combines the outputs of different TNs can allow users to have access to relevant existing networks of actors. Furthermore, it can allow having direct contact with farmers so that they can communicate their needs. Older farmers may need an intermediary, e.g. an advisor to help them decide which knowledge is relevant and how they can implement it. Hence, it is important of having digitally trained advisors and other key intermediary actors. However, to be successful and more impactful, the content needs to be well-structured and the interface of such a digital platform needs to be strongly user-oriented.

### Post execution

The evaluation of uptake and use of new knowledge is methodologically challenging (Walker et al. 2010). Sustainability indicators that are used are limited and only measure participation and engagement in project activities (Norström et al. 2020). Whether or not users adopted or adapted the TN knowledge is difficult to assess and it is complex and context-specific. The outcomes of the study state that, the uptake of results by farmers is, for example, the consequence of many parameters, as farmers/foresters‘ choices are influenced by many factors. Moreover, the necessary time for the project’s results to be taken up by users is usually quite long and starts only when the project delivers its first results (Carneiro and Garbero 2018).

Currently, there are approaches to evaluate the societal impact of research projects. Two were developed in France: the Socio-economic Analysis of Impacts of Public Agronomic Research (ASIRPA) and the Impact of Research in South approach (ImpreS *ex-ante*). Those two methodologies can represent a valid strategy to measure the impact; however, they are based on standardized case studies and on a long time scale and not yet applied in evaluating TNs (Joly et al. 2015; Blundo-Canto et al. 2018). In addition to these two approaches, the “Guidelines for Evaluation of Innovation in Rural Development Programmes” were developed by the European Network for Rural Development (ENRD) and the European Helpdesk for Rural Innovation (COM 2017a). The guidelines are meant to evaluate the performance of rural development programs of Member states. Still, their application should be considered appropriate for the TNs evaluation, since one of the targets of these guidelines is the EIP-AGRI and the OGs as well. More recently, the H2020 LIAISON project developed practice-ready tools for impact assessment and evaluation of any project/initiative. These tools aim to evaluate, monitor, improve and assess the impact of any interactive innovation process (Macken-Walsh et al. 2021). Future TNs should be encouraged to examine their interactive innovation process through the lens of the mentioned tools. This will help them to prepare for the selection of appropriate strategies for their particular activity being impact assessed.

Even though these methodologies can be applied, the inputs collected from the expert groups showed that evaluating a TN requires numerous resources (human, time, costs) and strategic planning. What was pointed out as an important step is that a good evaluation strategy at the beginning of the project is necessary to make it more impactful. Furthermore, funding schemes may be allocated by national and regional departments to enhance and to maintain TNs platforms running. An additional benefit is that an alive platform provides access to outputs also after the project is finished. This should allow long-term visibility and increase the potential of key intermediaries actors such as advisors and educators bringing the TN outcomes to the attention of farmers/foresters. Furthermore, the national/regional government can facilitate and financially support the mapping of local networks. Enhancing the collaboration of existing networks allows TNs outcomes to be transferred to users and hence broaden the outreach and uptake of their results.

## Conclusion

The MAA in TNs for agriculture and forestry innovation is overall a well understood and applied concept. This study highlighted the strengths and weaknesses of the MAA in three different phases of the TN projects: the conceptualisation, the implementation and the post-execution phase.

It was observed in the conceptualisation phase that the process of actor selection can be strongly influenced by previous cooperation experiences. This can lead to a lack of involvement of some new and innovative actors in TN consortia. A TN advisory board and a facilitator can strengthen farmers’ and foresters’ engagement through participatory activities (e.g. F2F interviews, workshops and surveys). The person in charge of covering the role of a facilitator does not need to have a technical level of expertise about the TN topic, although a sufficient level of competence in facilitating the MA knowledge exchange is needed.

Furthermore, it was observed that having an engagement strategy since the conceptualisation phase is essential to optimise knowledge exchange to ensure the uptake and exploitation of TNs results. The organization of participatory activities (e.g. farm demonstrations, peer-to-peer exchange and focus groups) and the creation of an interactive digital platform may improve the exchange between innovative and more conservative farmers in a broader geographical range. Additionally, the link within OGs can be boosted in each TN.

Lastly, the evaluation of knowledge uptake is still very challenging because there is no budget allocation for a post-execution or long-term evaluation. At the moment, several indicators have been used as tools to show the participation and engagement of users in TNs. However, they do not show whether users benefit from TN outputs. As such, further reflections are still needed in developing methodologies and indicators that allow having a long-term view. Involving heterogeneous actors in the early stages is essential to ensure the highest level of results uptake. Connecting partners through the share of common interests benefit the uptake of the results, as well as the TN’s impact. Therefore, this last post-execution stage requires sufficient resources as well as strategic planning.

A fully operational and effective MAA allows TNs to contribute to accelerating innovation in agriculture and forestry. Besides, more efficient, sustainable and impactful TNs can contribute to a well-functioning AKIS at local, national and European levels. As such, the information flow and the sharing of knowledge between AKIS key actors are strengthened.

## Supplementary Information

**Additional file 1.** **Annex I.** Questionnaire for the face-to-face interviews.

## Data Availability

Not applicable.

## Footnotes

1 https://euraknos.eu/.

2 https://liaison2020.eu/.

3 The coordinators of the missing TNs were present at the workshop in Budapest to provide their inputs and validate the results of the survey.

4 www.mentimeter.com.

5 www.polleverywhere.com.

6 https://www.mural.co/.

7 The following tools were used to ask questions and create an interactive atmosphere with participants. The online survey systems Mentimeter and PollEverywhere were used to ask participants their opinion on the specific objectives of the CEV. In addition, the application MURAL was used as a replacement for a post-it board/flip-over chart.

8 The adopter categorization is based on the work of Rogers (1958).

## References

[CR1] Blundo-Canto G, Triomphe B, Faure G, Barret D, De Romemont A, Hainzelin E (2018) Building a culture of impact in an international agricultural research organization: Process and reflective learning. Oxford University Press. 10.1093/reseval/rvy033

[CR2] Brunori G (2020). Agricultural and food economics: the challenge of sustainability. Agric Food Econ.

[CR3] COM (2012) Communication from the commission to the European parliament and the council on the European Innovation Partnership ‘Agricultural Productivity and Sustainability’

[CR4] COM (2013) Regulation (EU) No 1306/2013 of the European Parliament and of the Council of 17 December 2013 on the financing, management and monitoring of the common agricultural policy and repealing Council Regulations (EEC) No 352/78, (EC) No 165/94, (EC) No 2799/98. Off J Eur Union

[CR5] COM (2014) Guidelines on programming for innovation and the implementation of the EIP for agricultural productivity and sustainability

[CR6] COM (2017a) Guidelines evaluation of innovation in rural development programmes. In: Directorate-general for agriculture and rural development – Unit C.4 (2017): Guidelines. Evaluation of innovation in rural development programmes 2014–2020., (November)

[CR7] COM (2017b) Synergies between framework programmes for research and innovation and European structural and investment funds - contributing to the interim evaluation of Horizon 2020. 10.2777/403680

[CR8] Calliera M (2021). Multi-actor approach and engagement strategy to promote the adoption of best management practices and a sustainable use of pesticides for groundwater quality improvement in hilly vineyards. Sci Total Environ.

[CR9] Carneiro B, Garbero A (2018). Supporting impact with evidence: a content analysis of project completion reports. J Dev Stud.

[CR500] COM (2020) EUROPE 2020 A European strategy for smart, sustainable and inclusive growth. https://eur-lex.europa.eu/legalcontent/EN/TXT/PDF/?uri=CELEX:52010DC2020&from=EN

[CR10] Coniavitis E, Järnefelt C, Wojewoda N (2005). Involving the students: outcomes and experiences from the participation of the board of European students of technology in the thematic network E4. Eur J Eng Educ.

[CR200] Contini C, Marotta G, Torquati B (2020) Multi-actor approaches to implement cooperative strategies and value chains based on sustainability. Agri Food Econom. 10.1186/s40100-019-0147-3.

[CR11] Dogliotti S (2014). Co-innovation of family farm systems: a systems approach to sustainable agriculture. Agric Syst.

[CR13] EIP-AGRI (2016) Thematic networks under Horizon 2020 compiling knowledge ready for practice

[CR14] EURAKNOS (2020) Explorer ’ s guide to thematic networks how to design and implement thematic networks. Ghent, Belgium

[CR205] FAO (2018) FAO's work on Agricultural innovation. http://www.fao.org/3/CA2460EN/ca2460en.pdf

[CR15] Fieldsend AF (2020). Organisational Innovation Systems for multi-actor co-innovation in European agriculture, forestry and related sectors: Diversity and common attributes. NJAS Wageningen J Life Sci.

[CR16] Fieldsend AF (2021). ‘Sharing the space’ in the agricultural knowledge and innovation system: multi-actor innovation partnerships with farmers and foresters in Europe. J Agric Educ Ext.

[CR17] Frow P (2015). Managing co-creation design: a strategic approach to innovation. Br J Manag.

[CR18] Ingram J (2019). (2020) How do we enact co-innovation with stakeholders in agricultural research projects? Managing the complex interplay between contextual and facilitation processes. J Rural Stud.

[CR19] Joly PB (2015). ASIRPA: A comprehensive theory-based approach to assessing the societal impacts of a research organization. Res Eval.

[CR20] Kiptot E (2006). Sharing seed and knowledge: Farmer to farmer dissemination of agroforestry technologies in western Kenya. Agrofor Syst.

[CR21] Klerkx L (2017). Replication and translation of co-innovation: the influence of institutional context in large international participatory research projects. Land Use Policy.

[CR22] Lundsgaarde E, Keijzer N (2019). Development cooperation in a multilevel and multistakeholder setting: from planning towards enabling coordinated action?. Eur J Dev Res.

[CR23] Lundvall B-Å (2016) From user producer interaction to national systems of innovation, the learning economy and the economics of hope

[CR201] Macken-Walsh A (2016) Governance, partnerships and power In: Shucksmith M, Brown DL (eds) International handbook of rural studies, Routledge. https://www.researchgate.net/publication/307149659_Governance_partnerships_and_power

[CR24] Macken-Walsh Á (2019). Multi-actor co-design of extension interventions: paradoxes arising in three cases in the Republic of Ireland. J Agric Educ Ext.

[CR25] Macken-Walsh Á et al (2021) Impact assessment and evaluation tools - H2020 LIAISON project

[CR26] Madureira L et al (2019) Actors, roles and interactions in agricultural innovation networks: the case of the Portuguese cluster of small fruits. In: Smart innovation, systems and technologies, pp 42–49. 10.1007/978-3-319-92102-0_5

[CR600] Matthews A (2020) The new CAP must be linked more closely to the UN Sustainable Development Goals. Agri Food Econom. 10.1186/s40100-020-00163-3.

[CR27] Norström AV (2020). Principles for knowledge co-production in sustainability research. Nat Sustain.

[CR28] Rogers EM (1958) Categorizing the adopters of agricultural practices. Rural Sociol 346–354

[CR29] Rose DC (2016). Decision support tools for agriculture: Towards effective design and delivery. Agric Syst Authors.

[CR30] Rose DC (2021). Agriculture 4.0: Making it work for people, production, and the planet. Land Use Policy.

[CR31] SCAR AKIS (2019) Preparing for future akis in Europe - 4 th report of the strategic working group on agricultural knowledge and innovation systems

[CR32] Schneider F (2009). Social learning processes in Swiss soil protection—the ‘from farmer—to farmer’ project. Hum Ecol.

[CR33] Schwarz G, Vanni F, Miller D (2021). The role of transdisciplinary research in the transformation of food systems. Agric Food Econ.

[CR34] Sewell AM (2017). Using educational theory and research to refine agricultural extension: affordances and barriers for farmers’ learning and practice change. J Agric Educ Ext.

[CR37] Šūmane S (2018). Local and farmers’ knowledge matters! How integrating informal and formal knowledge enhances sustainable and resilient agriculture. J Rural Stud.

[CR35] Van Oost I et al (2017) The role of thematic network in EU agricultural innovation

[CR203] von Münchhausen S, Biró, Sz, Fieldsend A, Häring AM, Mayr S, Rønningen K (2019) Identification of key challenges and information needs of those enabling and implementing interactive innovation projects within the EIP-Agri. Presented at the 24th European Seminar on Extension (and) Education. 18-21 June 2019, Acireale, Italy. https://www.crea.gov.it/documents/68457/0/European+Seminar+on+Extension+and+Education+%285%29.pdf/0aa24566-c206-67ad-cdda-aead0c5e57c8?t=1606900561856

[CR36] Walker T, Ryan J, Kelley T (2010). Impact assessment of policy-oriented international agricultural research: evidence and insights from case studies. World Dev.

[CR207] Wielinga E, Geerling-Eiff FGE (2009) Networks with free actors 2009. 10.3920/978-90-8686-688-5

