# Supplementary material for: The multi-actor approach in thematic networks for agriculture and forestry innovation
Source: Agric Food Econ. 2022 Jan 17;10(1):3. doi: 10.1186/s40100-021-00209-0 (PMC8761514; doi:10.1186/s40100-021-00209-0)
Supplement: Supplementary file 1 — Additional file 1. Annex I. Questionnaire for the face-to-face interviews. [file 40100_2021_209_MOESM1_ESM.docx]

**Annex I**

**Questionnaire for the face-to-face interviews**

*Preliminary questions (Skype with the coordinator)*

1. What is the definition of MA in your TN?

2. Can we look at the multi-actor strategy?

3. How was the multi-actor strategy described in the TN proposal?

4. Do you have contact with the persons of the different groups of actors a) from the consortium group b) outside the consortium group (endorsers) c) outside the project who could answer these specific questions about MAA?

5. Did the MA strategy differ during the project progress from what was described in the proposal? (Y/N)

6. Who are the actors in the multi-actor group? (academics, policy-makers, advisors, consumers, farmers, facilitators. end-users)

7. What types of actors are reached by the consortium members (2nd level target group) ? (academics, policy-makers, advisors, consumers, farmers, facilitators. end-users)

8. How frequently do the actors of the consortium meet during the TN project? (numeric data)

9. What is the share of farmers (list of possible answers: number of involved farmers/ total number of stakeholders in the consortium)?

10. Which type of farmers was involved in the thematic network (list of possible answers: early adopters, multiplicators, innovators or farmers in a special region with special issues).

11. How many farmers of each type (early adopters, multiplicators, innovators or farmers in a special region with special issues) were on board? (numeric data)

12. Was it a more scientific project or a project that has emerged from challenges met by a group of farmers? (2 POSSIBLE ANSWERS: research and policy-driven projects/ demand-driven projects)

13. Co-creation of knowledge

14. What are the types of actors involved in each MA meeting/TN work?

15. (academics, policy-makers, advisors, consumers, farmers, facilitators. end-users, others)

16. Were you missing any type of actor (academics, policy-makers, advisors, consumers, farmers, facilitators. end-users) in your project?

17. Why was each type of actor approached? (best practices)

18. How was each type of actor approached? (best practices)

19. Why was each type of actor interested in joining (motivation)?

20. Were they involved in any other TN? (Y/N)

21. What were the barriers (e.g. stakeholder fatigue)?

22. How did they contribute (2 POSSIBLE ANSWERS. create content or only use as end-user)?

23. If some contents/ or materials were created, what type of content/ materials has been disclosed? (list of possible content/ material to be together defined)

24. Did new topics emerge from some MA processes (such as from peer-to-peer learning, collective thinking ?) (Y/N)

25. From when to when (start to finish/ map in time; 3 possible answers: beginning of the project, mid-term, or termination phase)?

26. Did the initial expectations have changed during the project/ after the project? (Y/N)

27. If yes, why did the initial expectations have changed throughout the project lifetime?

28. How many links were you able to make to other MA projects and groups in the EIP-Agri landscape (focus groups, operational groups, rural development networks,...) (numeric data)

*Subsidiary questions (if we have the time to raise these questions)*

1. How would you describe this process (main difficulties and suggestions/actions taken to facilitate this process)?

2. What have been the main reasons for these linkages (e.g. use of it in future projects, advisory services etc).

3. What is the geographical relevance of your TN? (are all relevant areas covered with regards to the TN agricultural topic?) (Y/N)

4. What communication channels have been mostly used and developed between all actors (consortium communication) and throughout the timeline?

5. Involvement of stakeholders

6. Has the involvement of actors changed over the project? (Y/N)

7. How the involvement of actors changed over the project?

8. Why do you think the involvement of actors has changed over the project?

9. Are meetings organized with farmers? (Y/N)

10. If yes, how often? (numeric data)

11. How many farmers attend these meetings? (numeric data)

12. Is there any impact indicator/evaluation method to measure the impact of the meetings? (Y/N)

13. If yes, what impact indicators did you use? (example: quantitative indicators such as the number of participants, evaluation grade, or more qualitative such as satisfaction survey, others ?)

14. How did the thematic network keep farmers and other actors engaged in meetings? (best practices)

15. Lessons learned from experienced projects and how you would approach things (differently or not) when you will start a new TN? (best practices)

*Subsidiary questions (if we have the time to raise these questions)*

1. What were the benefits for each type of actor for being involved in the project?

2. Which results/outputs are available? How did each type of actor benefit from the outputs of the project in the short, medium and, long term?

3. MA structure and sustainability of the TNs

4. Is the thematic network sustained beyond the life of the funding? (Y/N)

5. If yes, how is this sustainability ensured? (are there any informal operational groups that have continued beyond the life of the thematic network? Focus groups? Others ?)

6. What recommendations would you make in order to better ensure the sustainability of the TNs ? (best practices)

**Online survey**
